# Treating Exudative Pleurisy Accompanied by Felty Syndrome in an Older Patient With Advanced Rheumatoid Arthritis

**DOI:** 10.7759/cureus.37270

**Published:** 2023-04-07

**Authors:** Kanako Nonaka, Shota Watanabe, Chiaki Sano, Ryuichi Ohta

**Affiliations:** 1 Family Medicine, Shimane University Faculty of Medicine, Izumo, JPN; 2 Community Medicine Management, Shimane University Faculty of Medicine, Izumo, JPN; 3 Community Care, Unnan City Hospital, Unnan, JPN

**Keywords:** immunosuppressive agents, felty syndrome, exudative pleurisy, rheumatoid arthritis, rheumatoid vasculitis

## Abstract

Advanced rheumatoid arthritis (RA) is complicated by extra-articular manifestations such as small- and medium-sized vasculitis, pulmonary fibrosis, and pleurisy. The clinical course of the disease is refractory and critical. Treating advanced RA with multiple extra-articular manifestations is challenging. Here, we report a case of advanced RA in a 75-year-old man with exudative pleurisy and Felty syndrome. Treatment should be initiated promptly while paying attention to the possibility of infection as a differential diagnosis of exudative pleurisy because of the drastic change in the patient's condition due to disease progression. In addition, appropriate treatment is required to differentiate between Felty syndrome and malignant diseases. In older patients with RV complicated by pleurisy and Felty syndrome, starting steroids and immunosuppressive agents is crucial when conducting a thorough examination and considering the rapid progression of symptoms.

## Introduction

Advanced rheumatoid arthritis (RA) is associated with several complications and poor disease control. Anemia occurs most frequently in patients with advanced RA [1,2]. Among patients with advanced RA and anemia, Felty syndrome is rare but critical and causes splenomegaly and leukopenia [3]. The incidence of exudative pleurisy in patients with advanced RA is approximately 29% [4]. The chest radiographs incidentally detected asymptomatic pleural thickening and small pleural effusions. In cases of moderate-to-severe symptomatic exudation, pleurocentesis should be performed to investigate pleurisy due to tuberculosis, cancerous pleurisy, or other collagen diseases [4].

The co-existence of Felty syndrome and exudative pleurisy is challenging to treat. Advanced RA is complicated and fatal because of exudative pleurisy and myelosuppression caused by Felty syndrome and requires prompt treatment [5]. However, the underlying mechanism of Felty syndrome remains unclear, and it is difficult to distinguish it from other diseases [6]. We encountered an elderly patient with advanced RA complicated by acute-onset pleurisy and Felty syndrome. We treated the complications of advanced RA in frail elderly patients, excluded serious illnesses, and provided diagnostic treatment. Concrete treatment methods for the critical complications of advanced RA are discussed in this case report.

## Case presentation

A 75-year-old male patient presented to our hospital with a chief complaint of fatigue and fever. Two months before admission, when he had an acute onset of dyspnea and sputum, he was healthy. The patient was transferred to a tertiary hospital, diagnosed with bacterial pneumonia, and treated with ceftriaxone (2 g/day) for one week. Symptoms were alleviated, and the patient was discharged. However, after discharge, the patient experienced a mild persistent fever and fatigue. His joints swelled two weeks before admission, and he experienced dyspnea while moving at home. The symptoms progressed, and the patient visited a rural community hospital for further investigation. The patient was diagnosed with RA 20 years ago and treated with oral prednisolone (7.5 mg/day) and subcutaneous abatacept (125 mg/week). The other medical history included hypertension and osteoporosis. Other medications included enalapril (5 mg/day) and minodronic acid (50 mg/day). He had no history of illness or travel within the past three months.

The vital signs at the visit were as follows: blood pressure, 93/69 mmHg; pulse rate, 116 beats/min; body temperature, 38.7°C; respiratory rate, 20 breaths/min; and oxygen saturation, 90% on room air. The patient was alert regarding time, place, and person. Physical examination revealed decreased light lung sounds, bilateral anterior and posterior cervical lymphadenopathy, and swelling, tenderness, and deformities of the hands, elbows, knees, and leg joints. No neurological abnormalities were observed. No obvious abnormalities were observed in the abdomen or skin. Laboratory tests showed a high C-reactive protein (CRP) of 10.55 mg/dL, a high erythrocyte sedimentation rate of 160 mm/h with normocytic anemia (hemoglobin, 7.2g/dL), and low neutrophil, lymphocyte, and platelet counts (Table 1).

**Table 1:** Initial laboratory data of the patient. eGFR: estimated glomerular filtration rate; CK: creatine kinase; CRP: C-reactive protein; TSH: thyroid-stimulating hormone; Ig: immunoglobulin; HCV: hepatitis C virus; SARS-CoV-2: severe acute respiratory syndrome coronavirus 2; HBs: hepatitis B surface antigen; HBc: hepatitis B core antigen; C3: complement component 3; C4: complement component 4; MPO-ANCA: myeloperoxidase antineutrophil cytoplasmic antibody; CCP: cyclic citrullinated peptide.

Parameter	Level	Reference
White blood cells	2.10	3.5–9.1 × 10^3^/μL
Neutrophils	34.2	44.0–72.0%
Lymphocytes	60.9	18.0–59.0%
Monocytes	4.3	0.0–12.0%
Eosinophils	0.0	0.0–10.0%
Basophils	0.6	0.0–3.0%
Red blood cells	2.46	3.76–5.50 × 10^6^/μL
Hemoglobin	7.5	11.3–15.2 g/dL
Hematocrit	23.6	33.4–44.9%
Mean corpuscular volume	95.8	79.0–100.0 fL
Platelets	16.2	13.0–36.9 × 10^4^/μL
Erythrocyte sedimentation rate	160	2–10 mm/hour
Total protein	8.4	6.5–8.3 g/dL
Albumin	1.8	3.8–5.3 g/dL
Total bilirubin	0.5	0.2–1.2 mg/dL
Aspartate aminotransferase	13	8–38 IU/L
Alanine aminotransferase	6	4–43 IU/L
Alkaline phosphatase	86	106–322 U/L
γ-Glutamyl transpeptidase	16	<48 IU/L
Lactate dehydrogenase	141	121–245 U/L
Blood urea nitrogen	15.1	8–20 mg/dL
Creatinine	0.57	0.40–1.10 mg/dL
eGFR	90.0	>60.0 mL/min/L
Serum Na	130	135–150 mEq/L
Serum K	4.7	3.5–5.3 mEq/L
Serum Cl	97	98–110 mEq/L
Serum Ca	8.3	3.5–5.3 mg/dL
Serum P	5.1	0.2–1.2 mg/dL
Serum Mg	2.0	1.8–2.3 mg/dL
Ferritin	472.8	14.4–303.7 ng/mL
CK	8	56–244 U/L
CRP	10.55	<0.30 mg/dL
TSH	1.69	0.35–4.94 μIU/mL
Free T4	1.4	0.70–1.48 ng/dL
IgG	3273	870–1700 mg/dL
IgM	888	35–220 mg/dL
IgA	267	110–410 mg/dL
IgE	1534	<173 mg/dL
HBs antigen	0.00	IU/mL
HBs antibody	316.2	mIU/mL
HBc antibody	0.00	S/CO
HCV antibody	0.00	S/CO
Syphilis treponema antibody	0.00	S/CO
SARS-CoV-2 antigen	Negative	Negative
Anti-nuclear antibody	40	<40
C3	103	86–164 mg/dL
C4	6	17–45 mg/dL
MPO-ANCA	<1.0	<3.5 U/mL
anti-CCP antibody	>500	<5 U/mL
Rheumatoid factor	407	<15 U/mL
Urine test		
Leukocyte	Negative	Negative
Nitrite	Negative	Negative
Protein	Negative	Negative
Glucose	Negative	Negative
Urobilinogen	normal	
Bilirubin	Negative	Negative
Ketone	Negative	Negative
Blood	Negative	Negative
pH	5.5	
Specific gravity	1.020	

**Figure 1:**
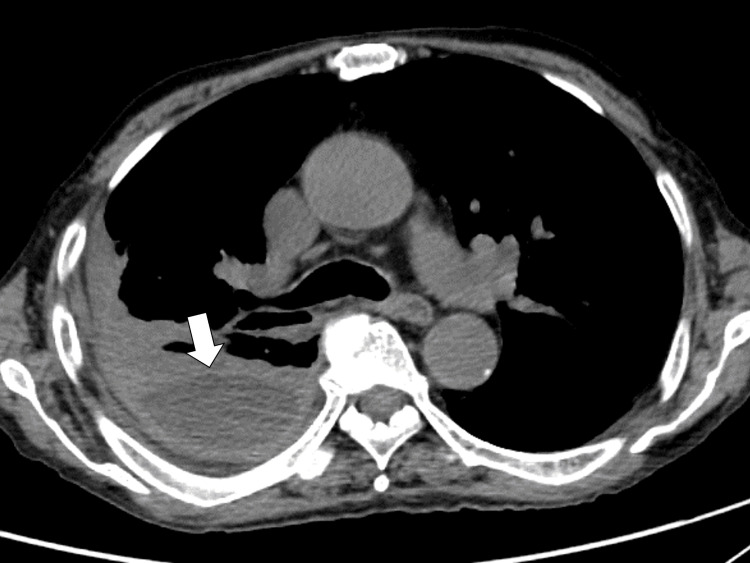
Chest CT showing right capsulated chest effusion (white arrow). CT: computed tomography.

Chest radiography revealed decreased opacity in the left chest. To investigate right chest effusion and systemic lymphadenopathy, chest computed tomography (CT) showed right capsulated chest effusion and lymphadenopathy (Figure 1).

**Figure 2:**
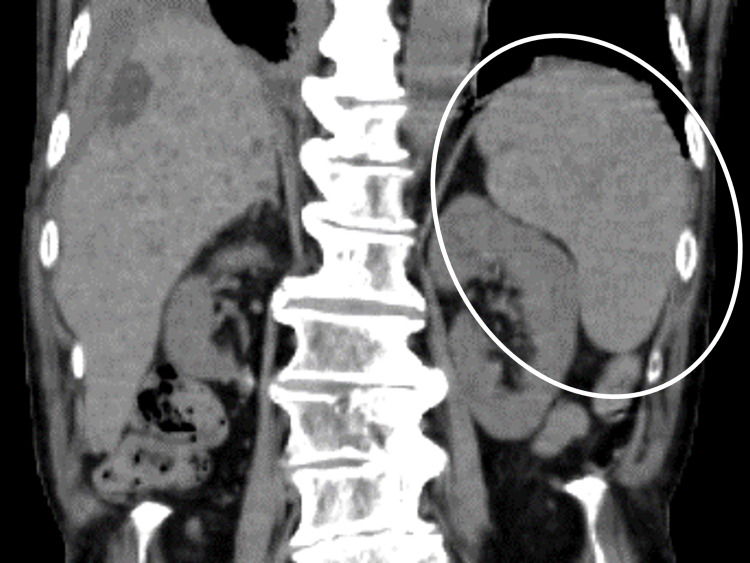
Abdominal CT showing splenomegaly (white circle). CT: computed tomography.

Abdominal CT revealed splenomegaly (Figure 2).

Investigation of the right pleural effusion showed exudative and highly inflammatory conditions with a pH of 7.216, lactate dehydrogenase level of 1243 IU/L, adenosine deaminase of 219.3 U/L, and glucose level of 20 mg/dL. 

Based on these clinical findings, bacterial empyema was diagnosed. Subcutaneous abatacept was discontinued, and the patient was treated with tazobactam/piperacillin (13.5 g/day). Bone marrow and right axillary lymph node biopsies showed no malignant findings, reactive swelling, or systemic inflammation. The patient was diagnosed with Felty syndrome because of advanced RA with inadequate control of splenomegaly, neutropenia, and thrombocytopenia. 

However, the patient’s fever and fatigue persisted after treatment, and blood and pleural effusion cultures were negative for bacteria and fungi. Follow-up test results for pleural effusion did not change under inflammatory conditions. Infectious causes cannot be completely ruled out due to the immunosuppressive reactions caused by Felty syndrome. However, the patient’s persistent inflammatory condition and swelling in multiple joints were considered extra-articular symptoms of advanced RA. On day 6 of admission, after a discussion with the patient and family, the patient was treated with prednisolone of 30 mg/day (0.5 mg/kg). 

On day 10 after admission, his fever disappeared. A follow-up laboratory test showed improvement in hemoglobin level, neutropenia, and thrombocytopenia, accompanied by improvement in joint findings and the inflammatory marker CRP. On day 21, the patient’s symptoms resolved, and he was followed up in the outpatient department. In the outpatient department, the prednisolone dose was tapered to 10 mg/day, which resulted in joint pain and fever. Subcutaneous tocilizumab (162 mg) was added every two weeks and alleviated the symptoms [7]. 

## Discussion

This case demonstrates the challenges in treating the complications of advanced RA in older patients. The differentiation between infections caused by immunosuppression and rheumatic complications from advanced RA depends on the clinical course. The vague diagnosis of Felty syndrome can be challenging; therefore, shared decision-making is integral to the planning and administering of comprehensive treatments for older patients with rheumatic diseases.

Considering the patient’s vital signs, the initial presentation, in this case, was similar to that of sepsis. The patient was treated empirically with antibiotics. However, the patient's clinical course was not typical of sepsis because the condition did not change with persistent fever and fatigue. The possibility of a bacterial infection dropped because the patient’s infection could not be alleviated or exacerbated [8]. Although the possibility of viral and fungal infections remained based on blood and pleural effusion cultures, we initiated prednisolone treatment for the tentative diagnosis of RA-related pleuritis accompanied by Felty syndrome [9]. Eventually, the prednisolone treatment was effective, and the patient’s symptoms disappeared. In immunocompromised patients, ruling out infections is difficult when they have a fever because delayed infection treatment may be fatal [10]. Thus, as shown in this case, immunosuppressed patients with autoimmune diseases should be treated with antibiotics before the complications caused by autoimmune diseases are diagnosed.

A vague diagnosis of Felty syndrome can also be challenging. Felty syndrome is a triad of RA, neutropenia, and splenomegaly that usually develops after long-term RA (at least 10 years) [11]. It is present in <1% of patients with RA [6]. RA in Felty syndrome generally has severe extra-articular manifestations, often complicating infections due to neutropenia and resulting in a poor prognosis [5]. The cause of Felty syndrome is unknown but has been found to occur primarily in patients with long-standing active RA who test positive for rheumatoid factor or anticitrullinated peptide, as in this case [12]. However, there is no specific diagnostic test for Felty syndrome, and other causative conditions, such as malignancy or myelofibrosis, should be ruled out. Although myelosuppression does not occur in malignant RA, it does occur when complicated by Felty syndrome [5].

The treatment of neutropenic fever in patients with advanced RA should be based on shared decision-making. Neutropenia (absolute neutrophil count <2000/µL) is present in all patients with Felty syndrome and is asymptomatic unless any infection occurs. Splenomegaly is present in 90% of patients with Felty syndrome and can be confirmed by palpation, ultrasonography, or scintigraphy [6]. The average spleen weight in the Felty syndrome group was four times the normal [6]. However, splenomegaly may also occur in Felty syndrome patients without neutropenia. Diagnosis may also be difficult in patients with Felty syndrome who develop an infection. White blood cells may be normal or elevated, and neutropenia may recur after successful infection treatment [13]. Sharing details of the patient's condition with him and his family could make the diagnosis and treatment processes more effective [14]. Thus, shared decision-making is integral to administering comprehensive treatments to older patients with advanced RA complicated by severe adverse effects [15].

## Conclusions

The treatment of advanced RA complications in older patients is challenging. Differentiating infections caused by immunosuppression and rheumatic complications from advanced RA requires a comprehensive approach depending on the patient’s immunosuppressive conditions. The vague diagnosis of Felty syndrome can be challenging; therefore, shared decision-making is essential for advancing the comprehensive treatment of older patients with rheumatic diseases.
